# Disparities in All-Cause Mortality Beyond the Acute Phase of the COVID-19 Pandemic in the US

**DOI:** 10.1001/jamanetworkopen.2023.56869

**Published:** 2024-02-20

**Authors:** Kaitlyn M. Berry, Andrew C. Stokes, Keeley J. Morris, Rafeya V. Raquib, Elizabeth Wrigley-Field

**Affiliations:** 1 Division of Epidemiology and Community Health, University of Minnesota School of Public Health, Minneapolis; 2 Minnesota Population Center, University of Minnesota, Minneapolis; 3 Department of Global Health, Boston University School of Public Health, Boston, Massachusetts; 4 Department of Sociology, University of Minnesota, Minneapolis

## Abstract

This cross-sectional study examines all-cause mortality in the US between March 2018 and May 2023 by sex, race and ethnicity, metropolitan status, and region.

## Introduction

The disparate effects of the COVID-19 pandemic on mortality by race, ethnicity, and geography have been extensively documented.^1,2,3^ However, less is known about whether the pandemic has altered US mortality disparities beyond its acute phase. This study examines the evolution of all-cause mortality disparities along major demographic axes between March 2018 and May 2023.

## Methods

We obtained mortality data from the National Center for Health Statistics (NCHS)^4^ and population data from the US Census Bureau. We calculated annualized age-standardized death rates (ASDRs) across 5.25 years from March 1, 2018, to May 31, 2023, by sex, race and ethnicity (as defined by death certifiers and NCHS), metropolitan status, and region. We estimated and compared mortality rate ratios across 4 summary periods: before the pandemic (combining 2018 and 2019 due to stable mortality rates), the pandemic’s first year (largely before the vaccine), the pandemic’s second year (after widespread vaccine availability), and the postacute period (largely after the first Omicron wave); statistical tests were not performed. Analysis was performed using Stata and R. This cross-sectional study was deemed not human research by the University of Minnesota institutional review board and therefore to not require approval. We followed the STROBE reporting guideline. See the eAppendix and eTable in Supplement 1 for methodological details.

## Results

The Figure shows ASDRs from March 2018 to May 2023 for the full US population. Mortality increased during both acute pandemic years before returning to near-prepandemic levels for most groups. The mortality increase was not equal across groups; it was largest for men, all minoritized racial and ethnic groups, nonmetropolitan areas, and the South. These changes created shifts in death rate ratios during the acute pandemic years (Table). A notable change was the increase in the American Indian or Alaska Native vs White mortality disparity from 1.02 (1.01-1.03) to 1.20 (1.18-1.21) or higher in both acute pandemic years. Despite these changes, death rate ratios during the postacute period generally returned to prepandemic levels. Exceptions include disparities by metropolitan status and region, with the disadvantage of nonmetropolitan areas (vs large metropolitan) remaining larger and the relative advantage of the West (vs Midwest) decreasing. In addition, while the Native Hawaiian or Other Pacific Islander rate ratio in the postacute period as a whole was similar to before the pandemic, their death rates uniquely increased in early 2023.

**Figure.**
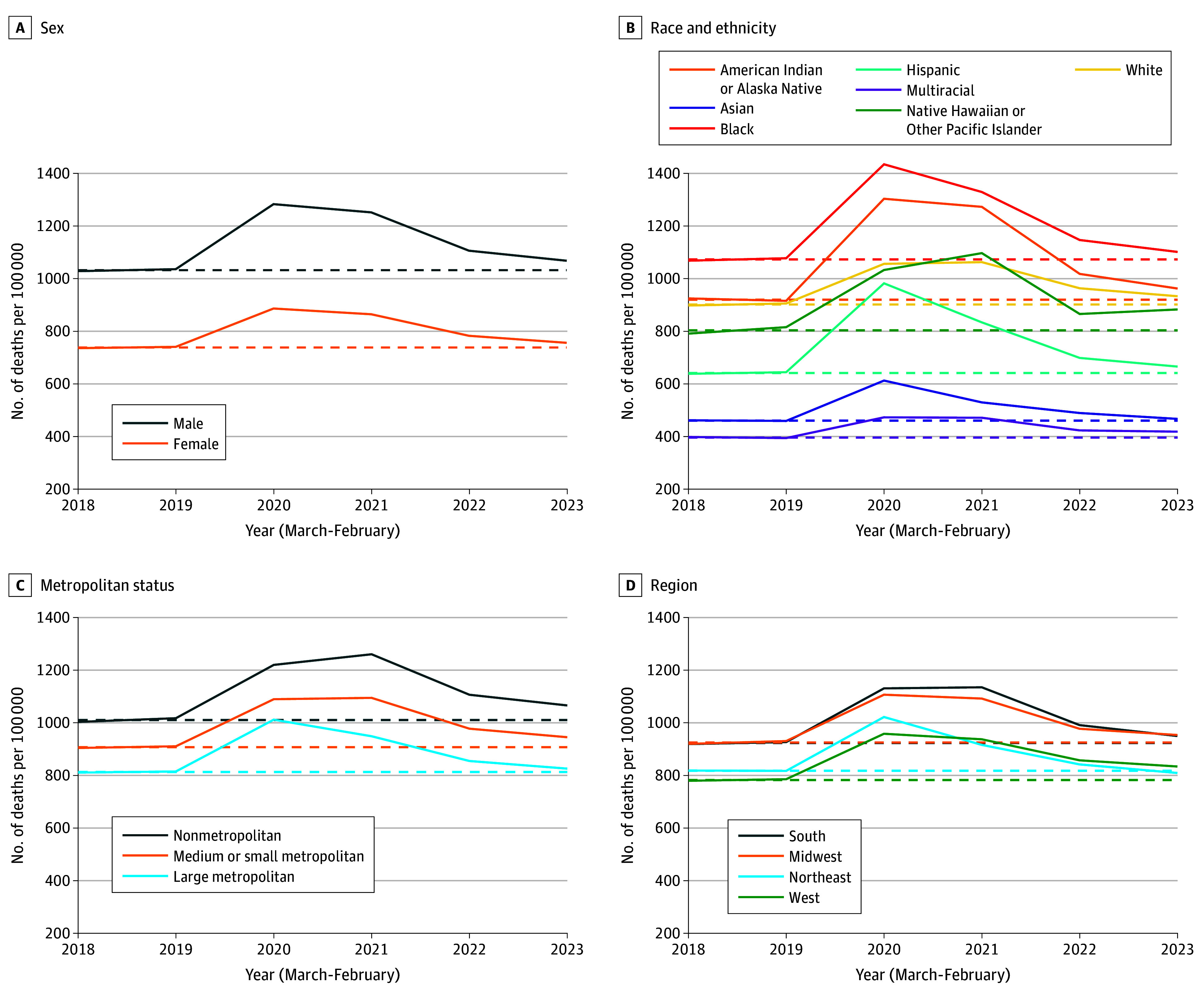
Age-Standardized Death Rates by Year and Demographic Characteristics Annual age-standardized death rates between 2018 and 2023 by sex (A), race and ethnicity (B), metropolitan status (C), and region (D). Years run from March to February of the subsequent year to align with the start of the COVID-19 pandemic. Death rates from 2023 include only 3 months (March, April, and May) due to lags in mortality data processing and are annualized to facilitate comparison with other years. Dashed lines indicate prepandemic (March 2018 to February 2020) death rates.

**Table.** Age-Standardized Death Rate Ratios Across 4 Periods by Demographic Characteristics

Comparison	Age-standardized death rate ratio (95% CI)			
	Prepandemic period (March 2018 to February 2020)^a^	Acute pandemic year 1 (March 2020 to February 2021)	Acute pandemic year 2 (March 2021 to February 2022)	Postacute period (March 2022 to May 2023)^b^
Sex				
Male vs female	1.40 (1.40-1.40)	1.45 (1.44-1.45)	1.45 (1.44-1.45)	1.41 (1.41-1.41)
Race and ethnicity				
American Indian or Alaska Native vs White	1.02 (1.01-1.03)	1.23 (1.22-1.25)	1.20 (1.18-1.21)	1.04 (1.02-1.05)
Asian vs White	0.51 (0.51-0.51)	0.58 (0.58-0.58)	0.50 (0.50-0.50)	0.50 (0.50-0.51)
Black vs White	1.19 (1.19-1.19)	1.36 (1.35-1.36)	1.25 (1.25-1.25)	1.18 (1.18-1.18)
Hispanic vs White	0.71 (0.71-0.71)	0.93 (0.93-0.93)	0.78 (0.78-0.79)	0.72 (0.72-0.72)
Native Hawaiian or Other Pacific Islander vs White	0.89 (0.87-0.91)	0.98 (0.95-1.01)	1.03 (1.00-1.06)	0.90 (0.88-0.92)
Multiracial vs White	0.44 (0.43-0.45)	0.45 (0.44-0.46)	0.44 (0.44-0.45)	0.44 (0.43-0.44)
Metropolitan status				
Nonmetropolitan vs large metropolitan	1.24 (1.24-1.25)	1.21 (1.20-1.21)	1.33 (1.32-1.33)	1.29 (1.29-1.30)
Small or medium metropolitan vs large metropolitan	1.12 (1.11-1.12)	1.08 (1.07-1.08)	1.15 (1.15-1.16)	1.14 (1.14-1.15)
Region				
South vs Midwest	1.00 (1.00-1.00)	1.02 (1.02-1.02)	1.04 (1.04-1.04)	1.01 (1.00-1.01)
Northeast vs Midwest	0.88 (0.88-0.89)	0.92 (0.92-0.93)	0.84 (0.84-0.84)	0.86 (0.86-0.86)
West vs Midwest	0.85 (0.84-0.85)	0.87 (0.86-0.87)	0.86 (0.86-0.86)	0.88 (0.87-0.88)

^a^ The prepandemic period combines 2018 (March 2018 to February 2019) and 2019 (March 2019 to February 2020) from the Figure into a single period.

^b^ The postacute period combines 2022 (March 2022 to February 2023) and 2023 (March to May 2023) from the Figure into a single period.

## Discussion

There has been a public debate about whether the US has “returned to normal” after the pandemic. Our study suggests that this has largely been the case with respect to disparities by gender, race and ethnicity, and region. Although mortality remains somewhat higher than before the pandemic, this additional mortality is largely proportional to 2018-2019 mortality with respect to major demographic comparisons. This continuity suggests that these disparities are persistent; even a pandemic-level mortality shock does not permanently alter them.

However, if the patterns identified here largely depict a “return to normal,” the “normal” that we have returned to is one of stark mortality disparities for groups such as Black individuals. This return to the high prepandemic level of mortality disparities reflects a failure to implement longer-term policy changes—such as universal paid sick leave, greater workplace rights to clean air, and stronger public health infrastructure—that might have further reduced these disparities once the acute pandemic ended.^5^

As an exception to our main result, we found that mortality in the postacute period was more heavily concentrated in nonmetropolitan areas than before the pandemic. This may reflect a combination of factors, including rural hospital closures, health care worker burnout, and increasing levels of polarization around vaccination and other preventive health care.^6^

Limitations include the use of provisional mortality data and the potential quality limitations of the 2020 US Census coverage. In addition, we were unable to include data prior to 2018 due to inconsistencies in the measurement of race and ethnicity.

## Notes

Supplement 1. **eAppendix.** Mortality Data **eTable.** Mortality Data Queries
eReferences.

Supplement 2.
Data Sharing Statement

## References

[1] Bassett MT, Chen JT, Krieger N. Variation in racial/ethnic disparities in COVID-19 mortality by age in the United States: a cross-sectional study. PLoS Med. 2020;17(10):e1003402. doi:10.1371/journal.pmed.1003402 33079941 PMC7575091

[2] Aschmann HE, Riley AR, Chen R, . Dynamics of racial disparities in all-cause mortality during the COVID-19 pandemic. Proc Natl Acad Sci U S A. 2022;119(40):e2210941119. doi:10.1073/pnas.2210941119 36126098 PMC9546535

[3] Lundberg DJ, Wrigley-Field E, Cho A, . COVID-19 mortality by race and ethnicity in US metropolitan and nonmetropolitan areas, March 2020 to February 2022. JAMA Netw Open. 2023;6(5):e2311098. doi:10.1001/jamanetworkopen.2023.11098 37129894 PMC10155069

[4] United States Department of Health and Human Services, Centers for Disease Control and Prevention, National Center for Health Statistics. Provisional multiple cause of death by single race, 2018-present. Published 2021. Accessed August 30, 2023. https://wonder.cdc.gov/mcd-icd10-provisional.html

[5] Bleser WK, Shen H, Crook HL, . Pandemic-driven health policies to address social needs and health equity. Health Affairs. March 10, 2022. Accessed January 3, 2024. https://www.healthaffairs.org/do/10.1377/hpb20220210.360906/

[6] Jones M, Bhattar M, Henning E, Monnat SM. Explaining the U.S. rural disadvantage in COVID-19 case and death rates during the Delta-Omicron surge: The role of politics, vaccinations, population health, and social determinants. Soc Sci Med. 2023;335:116180. doi:10.1016/j.socscimed.2023.116180 37713775 PMC10557078

