# Supplementary material for: Disparities in All-Cause Mortality Beyond the Acute Phase of the COVID-19 Pandemic in the US
Source: JAMA Netw Open. 2024 Feb 20;7(2):e2356869. doi: 10.1001/jamanetworkopen.2023.56869 (PMC10879948; doi:10.1001/jamanetworkopen.2023.56869)
Supplement: Supplement 1. — eAppendix. Mortality Data eTable. Mortality Data Queries eReferences. [file jamanetwopen-e2356869-s001.pdf]

Supplementary Online Content

Berry KM, Stokes AC, Morris KJ, Raquib RV, Wrigley-Field E. Disparities in all-cause mortality beyond the acute phase of the COVID-19 pandemic in the US. *JAMA Netw Open.* 2024;7(2):e2356869. doi:10.1001/jamanetworkopen.2023.56869

eAppendix. Mortality Data
eTable. Mortality Data Queries
eReferences.

This supplementary material has been provided by the authors to give readers additional information about their work.

eAppendix. Mortality Data

Data Source: CDC WONDER Provisional Mortality Statistics, 2018 through Last Month[1]

Rationale for Querying Approach: It is possible to sort CDC WONDER data by variables such as 10-year age groups, sex, race, ethnicity, urbanization, region, month, and year in one query. However, sorting in this way puts the data into smaller cells than needed for this study, resulting in unnecessary data suppression because death counts between 0 and 9 are censored. To limit data suppression, our approach was to submit multiple queries at every level of aggregation needed in the study. In total, we completed 42 queries which are summarized in the **eTable.**

eTable. Mortality Data Queries

<table>
<tr><th colspan="5">Provisional Mortality Statistics, 2018 through Last Month Request</th></tr>
<tr><th>Covariate:</th><th>Group Results By:</th><th>Select Location: Residence 2013 Urbanization:</th><th>Select Demographics: Hispanic Origin:</th><th>Select Time Period of Death:</th></tr>
<tr><td>Sex</td><td>Sex,
Ten-Year Age Groups</td><td></td><td></td><td rowspan="7">Separate files for each:

(1) March 2018 to February 2019

(2) March 2019 to February 2020

(3) March 2020 to February 2021

(4) March 2021 to February 2022

(5) March 2022 to February 2023

(6) March 2023 to May 2023</td></tr>
<tr><td rowspan="2">Race/Ethnicity</td><td>Single Race 6,
Ten-Year Age Groups</td><td></td><td>Not Hispanic or Latino</td></tr>
<tr><td>Ten-Year Age Groups</td><td></td><td>Hispanic or Latino</td></tr>
<tr><td rowspan="3">Metropolitan Status</td><td>Ten-Year Age Groups</td><td>Large metro = large central metro + large fringe metro</td><td></td></tr>
<tr><td>Ten-Year Age Groups</td><td>Small/Medium metro = medium metro + small metro</td><td></td></tr>
<tr><td>Ten-Year Age Groups</td><td>Nonmetro = micropolitan (nonmetro) + noncore (nonmetro)</td><td></td></tr>
<tr><td>Region</td><td>Residence Census Region,
Ten-Year Age Groups</td><td></td><td></td></tr>
</table>

Time Periods: Mortality data was extracted separately for six years running from March to February to match the start of the pandemic: (1) March 2018 to February 2019, (2) March 2019 to February 2020, (3) March 2020 to February 2021, (4) March 2021 to February 2022, (5) March 2022 to February 2023, and (6) March 2023 to May 2023. The sixth year only covers one quarter in order to provide an appropriate lag between the death reporting and data extraction. Additionally, the end of this period was chosen to align with the end of the national public health emergency declaration on May 11, 2023. Data was re-extracted by the research team during the revision stage in December 2023 to ensure that the more recent data were as complete as possible.

© 2024 Berry KM et al. *JAMA Network Open.*

To help summarize results for the eTable, we collapsed the two years from March 2018 to February 2020 into a “pre-pandemic period” and the 1.25 years between March 2022 to May 2023 into an “post-acute period.” March 2020 to February 2021 and March 2021 to February 2022 were considered the “acute pandemic period,” but were kept separate due to the differences in mortality rates and vaccine availability during the first and second years of the pandemic.

We did not report on any mortality patterns prior to March 2018 due to differences in the reporting of race and ethnicity in National Center for Health Statistics (NCHS) mortality data in earlier years (see below).

Racial Categorization: Race and Hispanic origin are reported separately on death certificates in accordance with Office of Management and Budget standards. The Provisional Multiple Cause of Death database currently provides data on deaths by six single race categories including (1) American Indian or Alaska Native (AIAN); (2) Asian; (3) Black or African American; (4) more than one race; (5) Native Hawaiian or Other Pacific Islander (NHOPI); and (6) White. Prior to 2018, death was only available by four single race categories including (1) American Indian or Alaska Native; (2) Asian or Pacific Islander; (3) Black; and (4) White. To provide mortality information by detailed racial categories, our analysis uses the death data from 2018 onward.

As with all death rates, there may be differences in the categorization of race and ethnicity in the numerators, which are listed on death certificates based on information from next of kin or on the basis of observation, and the denominators, which are based on individual self-reports that are subsequently processed by NCHS. These inconsistencies may create bias in death rates,[2] particularly for the multiple-race group.

Population Data

Data Source: The Population Estimates Program of the U.S. Census Bureau releases annual estimates of the US population for the nation, states, counties, state/county equivalents, and Puerto Rico. We used the Vintage 2020[3] and Vintage 2022[4] county resident population estimates to construct denominators for the age-standardized death rate (ASDR) calculations. Specifically, we used the “cc-est2020-all” file which contains “Annual County Resident Population Estimates by Age, Sex, Race, and Hispanic Origin: April 1, 2010 to July 1, 2020” and the “cc-est2022-all” file which contains “Annual County Resident Population Estimates by Age, Sex, Race, and Hispanic Origin: April 1, 2020 to July 1, 2022.”

2020 Population Estimates: Both data files contain population estimates for 2020. To avoid creating an artificial sharp break, we averaged the Vintage 2020 and Vintage 2022 population estimates for 2020.

Residence Urbanization: We used the 2013 NCHS’s Urban-Rural Classification Scheme for Counties to classify US counties and county-equivalents into six levels: (1) large central metro, (2) large fringe metro, (3) medium metro, (4), small metro, (5) micropolitan, and (6) non-core.[5] Consistent with prior work,[6,7] we condensed the data’s urban and rural classifications into 3 categories of residence: (1) large metropolitan, (2) medium and small metropolitan, and (3) nonmetropolitan areas. Large metropolitan areas refer to counties in metropolitan statistical areas with a population of 1 million residents or more. Medium and small metropolitan areas refer to counties in metropolitan statistical areas with a population between 50,000 and 999,999 residents. Nonmetropolitan areas refer to all other counties.

In 2022, the Census Bureau adopted the state of Connecticut’s nine Councils of Government as the county-equivalent geographic unit for the purpose of collecting, tabulating, and disseminating statistical data.[8] As a result, the Vintage 2022 county-level population estimates do not match the geographic boundaries of the previous eight Connecticut counties in the Vintage 2020 estimates. The NCHS Urban-Rural Classification

© 2024 Berry KM et al. *JAMA Network Open.*

Scheme for Counties has not yet been applied to the new Connecticut Councils of Government, making it difficult to accurately classify these new county-equivalents into large metropolitan, medium and small metropolitan, and nonmetropolitan areas. For Connecticut only, we used the Vintage 2020 estimates for 2018-2020 and the Vintage 2021 estimates for 2021. Thus, when extrapolating the data forward to 2023, the most recent estimate for Connecticut was from 2021 rather than from 2022.

Interpolation/Extrapolation: Starting with mid year population estimates from 2017-2022, we interpolated and extrapolated on the log scale to construct monthly estimates. We then took the mean population size for each period [Period 1: March 2018 to February 2019; Period 2: March 2019 to February 2020; Period 3: March 2020 to February 2021; Period 4: March 2021 to February 2022; Period 5: March 2022 to February 2023; Period 6: March 2023 to May 2023].

Age-Standardization Procedure

The US Census 2020 population was used as the standard population. For each demographic and temporal unit, age-specific death rates were calculated for ages 0-4, 5-14, 15-24, 25-34, 35-44, 45-54, 55-64, 65-74, 75-84, and 85+. Each ASDR was multiplied by the corresponding percentage of the standard population in that group, and then the components were added together to produce the ASDR. We also annualized mortality rates to account for the shorter length of period 6, which only covers March 2023 to May 2023.

We calculated the variance for our estimates of the ASDRs and rate ratios using the following approach:

Variance for Age-Standardized Death Rates (ASDRs)

The variance of the ASDRs were calculated using the method described in the CDC Vital Statistics of the United States Technical Appendix[9] with the following formula:

$$var(ASDR) = \sum \quad w_i^2 R_i^2 (\frac{1}{D_i})$$

Where *ASDR* is an age-standardized death rate, *i* is the age group (0-4, 5-14, 15-24, 25-34, 35-44, 45-54, 55-64, 65-74, 75-84, and 85+), w_i is the age specific weight based on the 2020 Census population age distribution, R_i is the age specific death rate, and D_i is the number of deaths used to calculate the age specific death rate.

Variance for Rate Ratios:

The variance of the rate ratios was calculated using the Delta method for calculating variance of ratios.

$$var(RR) = \frac{var(R_i) * R_r^2 + var(R_r) * R_i^2}{R_r^4}$$

This variance was then used to calculate the 95% confidence interval for the rate ratios with the following formula:

$$(\frac{R_i}{R_r}) \pm 1.96 * \sqrt{var(RR)}$$

© 2024 Berry KM et al. *JAMA Network Open.*

Where RR is the rate ratio, R_i is the ASDR being compared, and R_r is the referent group ASDR. Female was used as the referent group for sex comparisons. Non-Hispanic White was used as the referent group for race/ethnicity comparisons. Large metro was used as the reference group for metropolitan status comparisons, and Midwest was used as the reference group for region comparisons.

© 2024 Berry KM et al. *JAMA Network Open.*

eReferences.

1. United States Department of Health and Human Services (US DHHS), Centers for Disease Control and Prevention (CDC), National Center for Health Statistics (NCHS). Provisional Multiple Cause of Death by Single Race 2018 - present, on CDC WONDER Online Database, first released 12-6-2021. Data are compiled from data provided by the 57 vital statistics jurisdictions through the Vital Statistics Cooperative Program. National Center for Health Statistics Mortality Data on CDC WONDER. Published 2021. Accessed August 30, 2023. http://wonder.cdc.gov/mcd-icd10-provisional.html

2. Arias E, Heron MP, Hakes JK. *The Validity of Race and Hispanic-Origin Reporting on Death Certificates in the United States: An Update*. U.S. Department of Health and Human Services, Centers for Disease Control and Prevention, National Center for Health Statistics; 2016.

3. United States Census Bureau. *Methodology for the United States Population Estimates: Vintage 2020: Nation, States, Counties, and Puerto Rico- April 1, 2010 to July 1, 2020*.; 2021.

4. United States Census Bureau. *Methodology for the United States Population Estimates: Vintage 2022: Nation, States, Counties, and Puerto Rico- April 1, 2020 to July 1, 2022*.; 2022.

5. Ingram DD, Franco SJ. 2013 NCHS Urban-Rural Classification Scheme for Counties. *Vital Health Statistics*. 2014;2(166).

6. Elo IT, Hendi AS, Ho JY, Vierboom YC, Preston SH. Trends in Non-Hispanic White Mortality in the United States by Metropolitan-Nonmetropolitan Status and Region, 1990-2016. *Popul Dev Rev*. 2019;45(3):549-583.

7. Lundberg DJ, Wrigley-Field E, Cho A, et al. COVID-19 Mortality by Race and Ethnicity in US Metropolitan and Nonmetropolitan Areas, March 2020 to February 2022. *JAMA Netw Open*. 2023;6(5):e2311098.

8. Census Bureau. Change to County-Equivalents in the State of Connecticut. *Federal Register*. 2022;87:34235-34240. https://www.federalregister.gov/d/2022-12063

9. National Center for Health Statistics. *Vital Statistics of the United States: Mortality, 1999 Technical Appendix*.; 1999. https://wonder.cdc.gov/wonder/sci_data/mort/mcmort/type_txt/mcmort05/techap99.pdf

© 2024 Berry KM et al. *JAMA Network Open.*
