# Supplementary material for: Disparities in All-Cause Mortality Beyond the Acute Phase of the COVID-19 Pandemic in the US
Source: JAMA Netw Open. 2024 Feb 20;7(2):e2356869. doi: 10.1001/jamanetworkopen.2023.56869 (PMC10879948; doi:10.1001/jamanetworkopen.2023.56869)
Supplement: Supplement 2. — Data Sharing Statement [file jamanetwopen-e2356869-s002.pdf]

Data Sharing Statement

Berry. Disparities in All-Cause Mortality Beyond the Acute Phase of the COVID-19 Pandemic in the US. *JAMA Netw Open*. Published February 20, 2024.
doi:10.1001/jamanetworkopen.2023.56869

Data
Data available: Yes
Data types: Data (not involving human participants)
How to access data: Data for this project can be accessed at https://doi.org/10.17605/OSF.IO/32GRY
When available: With publication

Supporting Documents
Document types: Statistical/analytic code
How to access documents: Analytic code can be accessed at https://doi.org/10.17605/OSF.IO/32GRY
When available: With publication

Additional Information
Who can access the data: Anyone
Types of analyses: For any purpose
Mechanisms of data availability: Data are freely available
Any additional restrictions: none
